# Differential Effects of Art Therapy and Dance/Movement Therapy on Emotional and Somatic Regulation in Early Psychopathology: First-Episode Psychosis and Eating Disorders

**DOI:** 10.3390/brainsci16020211

**Published:** 2026-02-11

**Authors:** Annarita Vignapiano, Francesco Monaco, Claudio Malangone, Stefania Landi, Stefania Palermo, Naomi Gammella, Ilaria Pullano, Gaetano Pinto, Raffaele Malvone, Luigi Aruta, Luca Steardo, Giulio Corrivetti

**Affiliations:** 1 Department of Mental Health, ASL Salerno, 84131 Salerno, Italy; annarita.vignapiano@gmail.com (A.V.); fmonaco1980@gmail.com (F.M.); c.malangone@aslsalerno.it (C.M.); stefaniapalermo94@gmail.com (S.P.); naomigammella@gmail.com (N.G.);; 2 European Biomedical Research Institute of Salerno (EBRIS), 84121 Salerno, Italy; 3 Department of Health Sciences, University Magna Graecia of Catanzaro, 88100 Catanzaro, Italylucasteardo@gmail.com (L.S.J.); 4 Sport Science Faculty, University Giustino Fortunato, 82100 Benevento, Italy; l.aruta@unifortunato.eu

**Keywords:** creative expressive therapies, art therapy, dance/movement therapy, eating disorders, first-episode psychosis, emotional and somatic regulation

## Abstract

**Background:** Creative Expressive Therapies, including Art Therapy and Dance/Movement Therapy (DMT), are increasingly integrated as adjunctive interventions in the treatment of complex psychiatric conditions. However, comparative evidence regarding their differential effects across diagnostic groups remains limited. **Methods:** This exploratory quasi-experimental 2 × 2 factorial study compared Art Therapy and DMT, delivered as adjuncts to treatment as usual, in patients with first-episode psychosis (FEP) and eating disorders (EDs) (N = 36). Participants received ten weekly group sessions. Changes in perceived well-being, emotional tension regulation, and physical tension regulation were assessed at baseline and post-intervention using self-report measures. Data were analyzed using repeated-measures ANOVA and linear mixed-effects models. **Results:** Significant pre–post improvements were observed across all outcome domains, indicating a transdiagnostic effect of Creative Expressive Therapies. Differential response patterns emerged according to clinical profile and therapeutic modality. DMT was associated with relatively greater improvements in physical tension regulation in patients with EDs, whereas Art Therapy showed relatively greater effects on emotional tension regulation in patients with FEP. **Conclusions:** Within the limitations of an exploratory, non-randomized design and the use of non-validated outcome measures, the findings suggest modality-specific patterns of response to Creative Expressive Therapies. These results should be considered hypothesis-generating and support further investigation through adequately powered randomized controlled trials employing validated clinical and neurobiological outcomes.

## 1. Introduction

The treatment of complex psychiatric disorders, including psychotic disorders (particularly schizophrenia spectrum disorders) and eating disorders (EDs), requires a multidisciplinary approach integrating pharmacotherapy, psychotherapy, and psychosocial interventions [1]. Across both conditions, patients frequently present with marked disturbances in self-perception, emotional expression and regulation, as well as alterations in bodily experience and somatic awareness [2]. In EDs, distorted body image and a conflicted relationship between mind and body represent core psychopathological features that are often only partially addressed by verbally mediated psychotherapeutic approaches, underscoring the need for interventions that directly target embodied experience [3]. Similarly, in psychotic disorders, impairments in verbal communication and the high prevalence of negative symptoms in recent-onset psychosis such as affective flattening and social withdrawal frequently limit the effectiveness of purely verbal treatments [4]. These limitations highlight the clinical relevance of alternative, non-verbal expressive modalities capable of facilitating access to internal experiences and supporting psychosocial functioning [5]. In this context, art therapy integrated with mentalizing-oriented practices has been proposed as a feasible and clinically meaningful group intervention within Early Intervention in Psychosis services, with potential benefits for mentalizing capacity and social functioning [6].

Disturbances in embodied self-experience and bodily regulation have been independently documented across psychosis-spectrum conditions and eating disorders, suggesting a transdiagnostic alteration in body-based self-processing, interoception, and emotional–somatic integration [7,8,9,10].

### 1.1. Shared Disturbances in Embodied Self-Experience and Regulation

Although first-episode psychosis (FEP) and EDs are nosographically distinct conditions, converging theoretical and empirical models highlight shared disturbances in embodied self-experience, affect regulation, and the integration between bodily signals and emotional meaning. Both clinical populations frequently exhibit alterations in bodily awareness, heightened somatic tension, and difficulties in regulating affective states through verbal–symbolic means alone. In early psychosis, disruptions of self-boundaries, affective instability, and impaired symbolization limit access to verbal psychotherapeutic processes, particularly in the presence of negative symptoms. Similarly, EDs are characterized by distorted body representation, altered interoceptive processing, and a conflicted relationship between bodily sensations and emotional experience. From a dimensional and transdiagnostic perspective, these shared dysfunctions suggest that both groups may benefit from interventions targeting embodied and non-verbal regulatory processes. Accordingly, the present study does not aim to compare diagnostic categories per se, but rather to explore whether different creative expressive modalities differentially engage shared dimensions of emotional and somatic regulation across distinct clinical profiles.

### 1.2. Creative Expressive Therapies as Integrative Interventions

Within this framework, Creative Expressive Therapies (CETs), particularly Art Therapy and Dance/Movement Therapy (DMT), have received increasing attention as complementary interventions in mental health care. These approaches emphasize non-verbal expression and creative processes as primary means for exploring, expressing, and integrating emotional, cognitive, and bodily experiences [11]. Art Therapy provides a structured and containing medium through which patients can externalize internal experiences in symbolic and concrete forms, thereby reducing reliance on verbal articulation alone. In schizophrenia spectrum disorders, available evidence suggests that Art Therapy may be particularly effective in ameliorating negative symptoms and enhancing psychosocial functioning, whereas its impact on positive symptoms appears more limited [12,13]. In ED populations, art-based interventions have been shown to facilitate the exploration of poorly articulated emotional states, reduce state anxiety, and redirect attentional focus away from food- and weight-related preoccupations [14,15]. DMT directly engages bodily experience through movement, rhythm, posture, and interpersonal attunement, thereby targeting the somatic dimension of psychopathology. In EDs, DMT has been associated with improvements in body image, body awareness, and self-esteem, supporting a more adaptive and less judgmental relationship with the body in motion [16,17]. Art Therapy is included in United Kingdom national clinical guidelines for the treatment of psychosis [6,18,19]. Early pilot randomized controlled trials have reported improvements associated with Art Therapy across domains closely linked to brain function, including social and emotional awareness [18,20], negative symptoms, and self-referential and interpersonal attitudes [21]. These domains map onto neural systems implicated in psychosis, such as salience attribution, affect regulation, and self-processing networks. However, subsequent reviews have highlighted that, despite being perceived as meaningful and acceptable by both clinicians and service users, Art Therapy remains characterized by insufficiently standardized and theoretically grounded practice models. This limitation hampers integration with brain-based, cognitive, and mechanistic research frameworks and constrains the identification of neural targets or biomarkers of treatment response [22].

### 1.3. Literature Gap and Study Objectives

Although the existing literature supports the potential utility of both Art Therapy and DMT as adjunctive interventions, systematic reviews consistently identify substantial methodological limitations, including small sample sizes, heterogeneity of outcome measures, and a paucity of high-quality controlled studies [17]. Importantly, direct comparative investigations examining the differential effects of distinct CET modalities within well-defined clinical populations remain scarce. The present study seeks to address this gap by directly comparing the differential patterns of change associated with Art Therapy and Dance/Movement Therapy, delivered as adjuncts to treatment as usual, in patients with FEP and EDs. Specifically, the study examines changes in perceived well-being, emotional tension regulation, and physical tension regulation and explores whether therapeutic effects differ according to clinical profile and expressive modality.

Accordingly, the study aims to achieve the following:

evaluate within-group pre–post changes in perceived well-being, emotional tension regulation, and physical tension regulation;
compare the relative effectiveness of Art Therapy and DMT;
examine whether treatment effects differ as a function of clinical group (FEP vs. EDs).

### 1.4. Study Hypotheses

Based on existing theoretical models and empirical evidence, the following hypotheses were formulated:

**H1a:**  *Both Art Therapy and Dance/Movement Therapy will be associated with significant improvements across all outcome variables in both clinical groups.*

**H1b:**  *Dance/Movement Therapy will be associated with relatively greater improvements in patients with EDs, particularly with respect to improvements in physical tension regulation and perceived well-being, due to its direct engagement with embodied processes.*

**H1c:**  *Art Therapy will be associated with relatively greater improvements in emotional tension regulation and perceived well-being in patients with FEP, by providing a structured symbolic medium for emotional externalization.*

## 2. Methods

### 2.1. Study Design

The study adopted a quasi-experimental factorial 2 × 2 design with pre- and post-intervention assessments (T0 and T1). The design aimed to explore differential patterns of change associated with two adjunctive therapeutic interventions, Art Therapy and Dance/Movement Therapy (DMT), across two distinct clinical groups: patients with FEP and patients with EDs.

Both interventions were delivered as add-ons to treatment as usual (TAU). All participants continued to receive standard psychiatric care, including pharmacological treatment and routine psychosocial support, throughout the study period. No changes to TAU were introduced as part of the research protocol.

### 2.2. Participants

The total sample consisted of 36 patients recruited in Early Intervention in Psychosis and the Regional Eating Disorders Center of the Department of Mental Health, ASL Salerno (Italy).

Inclusion criteria were as follows:

- a primary diagnosis of FEP according to DSM-5 or ICD-11 criteria, or a diagnosis of EDs according to DSM-5 criteria;
- age between 14 and 20 years;
- a level of clinical stability deemed sufficient to allow participation in structured group-based interventions.

Exclusion criteria for both groups were as follows:

- a history of moderate to severe mental retardation or of neurological diseases;
- a history of alcohol and/or substance abuse in the last six months;
- current pregnancy or lactation;
- inability to provide informed consent to the residential inpatient treatments. FEPs with treatment modifications and/or hospitalization due to symptom exacerbation in the last three months were excluded. Participants were allocated to one of four study groups based on clinical availability and therapeutic suitability, rather than random assignments reported in Table 1. The non-randomized allocation reflects the pragmatic, real-world clinical setting in which the study was conducted.

### 2.3. Therapeutic Interventions

Art Therapy and Dance/Movement Therapy were delivered in parallel following a shared structural protocol:

Total duration: 10 sessions
Frequency: once weekly
Session length: 60 min per session

All sessions were delivered in a group format by licensed, certified therapists (a qualified art therapist and a qualified dance/movement therapist), each with specific clinical expertise in the treatment of the target disorders. Both interventions followed structured and manualized protocols, tailored to their respective modalities. Despite methodological differences, both approaches emphasized the non-verbal exploration and expression of emotional and bodily experiences, with the aim of enhancing emotional regulation, bodily awareness, and subjective well-being.

### 2.4. Outcome Measures

To assess clinically relevant constructs not fully captured by standard psychometric instruments, the study employed self-administered questionnaires specifically developed for this research and administered before and after the intervention. These ad hoc tools were designed to capture participants’ subjective experiences and process-related dimensions targeted by Creative Expressive Therapies, including perceived emotional state, physical and emotional tension, relaxation, cooperation, and quality of interaction during the sessions. Responses were recorded using a 5-point graphical Likert-type scale based on facial expressions, ranging from very negative to very positive states. This visually supported format was intended to facilitate comprehension, reduce cognitive load, and enhance engagement, allowing for the assessment of immediate and ecologically valid subjective changes associated with the interventions. Overall, the pre- and post-intervention questionnaires complemented standardized measures by providing fine-grained information on experiential and contextual aspects that are central to embodied and expressive therapeutic processes. These instruments were conceived as process-oriented measures capturing immediate subjective and embodied changes associated with Creative Expressive Therapies, rather than as symptom-based or diagnostic outcome measures. The dependent variables are reported in Table 2. Each ad hoc scale consisted of multiple items assessing the target construct, with responses scored on a 5-point Likert-type scale. For each outcome, total scores were computed by summing item responses, with higher scores indicating greater perceived well-being or more effective emotional or physical tension regulation. Although these instruments have not undergone formal validation, internal consistency was evaluated in the present sample and yielded acceptable to good reliability indices (Cronbach’s α ≥ 0.80). The use of ad hoc measures was motivated by the need to capture immediate, embodied, and process-related dimensions that are central to Creative Expressive Therapies but are not adequately represented by standard symptom-based instruments.

### 2.5. Statistical Analysis

All statistical analyses were performed using the R statistical computing environment (version 4.3.1; R Foundation for Statistical Computing, Vienna, Austria). All statistical tests were two-tailed, and the threshold for statistical significance was set at α = 0.05. Given the exploratory and naturalistic nature of the study, no a priori power analysis was conducted.

#### 2.5.1. Data Preparation and Preliminary Analyses

Data were initially screened for accuracy, completeness, and internal consistency. Composite scores for the dependent variables Perceived Well-Being (U1), Emotional Tension Regulation (U2), and Physical Tension Regulation (U3) were calculated by summing item scores for each scale for each participant at each assessment time point (baseline, S1; post-intervention, S10).

Missing data were handled using listwise deletion on an analysis-by-analysis basis, in order to preserve the internal consistency and interpretability of the statistical models applied.

#### 2.5.2. Reliability of the Measurement Instruments

The internal consistency of the ad hoc self-administered questionnaires was assessed using Cronbach’s alpha (α). Values of α ≥ 0.70 were considered indicative of acceptable reliability. All scales demonstrated good to excellent internal consistency, supporting their use in subsequent inferential analyses.

#### 2.5.3. Assessment of Statistical Assumptions

Prior to the application of parametric statistical models, key assumptions were evaluated. The normality of residual distributions was assessed using the Shapiro–Wilk test, while homogeneity of variance was evaluated through visual inspection of residual plots. No major violations were observed. Given the exploratory nature of the study and the limited sample size, interaction effects were a priori considered hypothesis-generating rather than confirmatory. No correction for multiple testing was applied. Accordingly, findings derived from interaction analyses are intended to inform future adequately powered randomized designs rather than to provide definitive evidence of differential efficacy.

## 3. Results

### 3.1. Sample Characteristics and Psychometric Properties

The overall sample consisted of 36 patients recruited from the Early Psychosis and Eating Disorders Centers. The sample showed a marked predominance of female participants, with 30 women (83.3%) and 6 men (16.7%), consistent with the epidemiological characteristics of the clinical populations under investigation. The internal consistency of the ad hoc self-administered scales was assessed using Cronbach’s alpha (α), yielding good to excellent reliability indices. Specifically, Perceived Well-Being (U1) demonstrated excellent internal consistency (α = 0.88), while Emotional Tension Regulation (U2) and Physical Tension Regulation (U3) showed good reliability (α = 0.84 and α = 0.81, respectively), supporting the psychometric adequacy of the instruments for subsequent inferential analyses. Sample characteristics and internal consistency indices for the outcome measures are reported in Table 3.

### 3.2. Overall Pre–Post Changes Associated with the Interventions

To test Hypothesis H1a, concerning the overall effectiveness of Creative Expressive Therapies irrespective of treatment modality and clinical diagnosis, repeated-measures ANOVA was conducted with Time (baseline, S1 vs. post-intervention, S10) as the within-subject factor. A significant main effect of Time was observed for all dependent variables, accompanied by very large effect sizes. For Perceived Well-Being (U1), mean scores increased from 28.58 ± 5.1 at baseline to 41.67 ± 5.4 post-intervention, F(1, 32) = 142.12, *p* < 0.001, η^2^*p* = 0.81. Emotional Tension Regulation (U2) also showed a significant increase, from 27.61 ± 5.4 to 38.08 ± 5.2, F (1, 32) = 118.45, *p* < 0.001, η^2^*p* = 0.78. Similarly, Physical Tension Regulation (U3) improved from 28.67 ± 5.8 at baseline to 39.06 ± 5.5 post-intervention, F (1, 32) = 105.73, *p* < 0.001, η^2^*p* = 0.76. Descriptive statistics and repeated-measures ANOVA results for all outcome variables are summarized in Table 4.

### 3.3. Differential Patterns of Change and Clinical Specificity

To examine differential treatment effects as a function of therapeutic modality and clinical group, linear mixed-effects models (LMMs) were estimated, including the Treatment × Clinical Group × Time interaction, with participants specified as a random intercept. The results of the linear mixed-effects models examining the Treatment × Clinical Group × Time interaction are reported in Table 5.

#### 3.3.1. Eating Disorders (H1b)

In line with Hypothesis H1b, a significant interaction emerged in favor of Dance/Movement Therapy in patients with EDs for Physical Tension Regulation (U3). The DMT × ED × Time interaction was statistically significant (β = 2.82, t = 1.98, *p* = 0.019), indicating a relatively greater magnitude of change in somatic regulation compared to Art Therapy. A further significant interaction in favor of DMT was observed for Perceived Well-Being (U1) (β = 3.09, t = 1.99, *p* = 0.049). Effect size analyses supported the clinical relevance of these findings. For Physical Tension Regulation (U3) in the ED group, Dance/Movement Therapy yielded a large effect size (d = 0.92), compared with a medium effect size for Art Therapy (d = 0.65). Effect size estimates by clinical subgroup and intervention are reported in Table 6.

#### 3.3.2. First-Episode Psychosis (H1c)

Consistent with Hypothesis H1c, results indicated a relatively greater change associated with Art Therapy in patients with FEP for Emotional Tension Regulation (U2). The Art Therapy × FEP × Time interaction was statistically significant (β = 1.57, t = 2.45, *p* = 0.026). Effect size estimation further supported the magnitude of this effect, with Art Therapy showing a large effect size for Emotional Tension Regulation (d = 0.84), compared with a medium effect size observed for Dance/Movement Therapy (d = 0.58) within the same clinical group. Effect size estimates by clinical subgroup and intervention are reported in Table 6.

### 3.4. Exploratory Analysis of Longitudinal Improvement Trends

This analysis was exploratory and descriptive in nature and aimed to visualize potential non-linear trajectories of change across treatment sessions rather than to test inferential hypotheses.

Exploratory analysis of the temporal progression of mean scores across treatment sessions revealed a non-linear pattern of clinical change. During the initial phase (sessions 1–3), improvements were not statistically significant (*p* > 0.05), suggesting an initial period of therapeutic latency. The intermediate phase (sessions 4–8) was characterized by a marked increase in clinical effectiveness, with statistically significant improvements (*p* < 0.01). In the final phase (sessions 9–10), treatment gains were maintained, with scores remaining significantly higher than baseline levels (*p* < 0.05). A summary of the temporal progression of treatment effects across intervention phases is presented in Table 7. No inferential statistical testing was performed for these phase-based comparisons.

## 4. Discussion

The present study explored patterns of change and clinical specificity of two Creative Expressive Therapies, DMT, integrated into treatment as usual in patients with FEP and EDs. Overall, both interventions were associated with significant pre–post improvements across all outcome domains, with large within-sample effect sizes observed for perceived well-being as well as emotional and physical tension regulation. Effect sizes should be interpreted as indicators of within-sample change in process-oriented outcomes rather than as estimates of clinical efficacy, given the exploratory nature of the study and the use of ad hoc self-report measures. As with other naturalistic pre–post designs, the present study is subject to potential sources of bias, including expectancy effects, selection bias, and regression to the mean. Consequently, observed changes may partially reflect these methodological factors rather than intervention-specific mechanisms.

### 4.1. Transdiagnostic Effectiveness of Creative Expressive Therapies

In line with Hypothesis H1a, both interventions were associated with significant improvements in perceived well-being, emotional tension regulation, and physical tension regulation, with large within-sample effects observed across outcome measures. These findings are consistent with previous evidence highlighting the potential of Creative Expressive Therapies to address emotional and embodied dimensions that are often insufficiently targeted by verbally mediated psychotherapies [11,17]. Recent integrative reviews further support the transdiagnostic impact of arts- and movement-based interventions on affective regulation and embodied functioning across heterogeneous psychiatric populations [23,24]. The non-linear temporal pattern observed in the present study, characterized by an initial phase of limited change followed by a peak of improvement during the intermediate treatment phase, is consistent with experiential learning models and theories of embodied reorganization. These frameworks emphasize the gradual consolidation of regulatory capacities through repeated sensorimotor and affective experiences rather than immediate symptom change [25,26]. Similar temporal trajectories have been reported in other body-oriented and arts-based interventions, suggesting that a minimum exposure period may be required for clinically meaningful embodied changes to emerge [27,28].

### 4.2. Specific Effectiveness of DMT in Eating Disorders

Consistent with Hypothesis H1b, DMT was associated with relatively greater effectiveness in patients with EDs, particularly with respect to improvements in physical tension regulation. This pattern is coherent with theoretical models conceptualizing EDs as conditions characterized by profound disturbances in bodily awareness, interoceptive processing, and embodied self-experience [10]. Movement-based interventions such as DMT may facilitate a reorganization of bodily experience by directly engaging proprioceptive and interoceptive processes, thereby supporting more adaptive regulation of somatic tension and bodily discomfort [17]. Emerging clinical evidence indicates that structured DMT protocols can enhance body awareness, mindfulness, and somatic regulation in populations marked by altered bodily self-experience [28,29]. In the present study, the observed improvement in perceived well-being among ED patients receiving DMT suggests that enhanced somatic regulation may extend beyond bodily domains and generalize to broader aspects of subjective well-being. This finding aligns with predictive processing and interoceptive models of emotion, which emphasize the close coupling between bodily regulation and affective experience [30,31]. Previous studies further suggest that movement- and body-oriented interventions may reduce alexithymia and enhance emotional awareness in individuals with severe EDs, complementing cognitively oriented treatments [27,32].

### 4.3. Specific Effectiveness of Art Therapy in First-Episode Psychosis

In accordance with Hypothesis H1c, Art Therapy was associated with relatively greater improvements in emotional tension regulation in patients with FEP. This finding can be interpreted in light of core psychopathological features of FEP, including difficulties in symbolization, affective instability, and vulnerability of self-boundaries [33,34]. By enabling the creation of a concrete and bounded external object, Art Therapy may provide a symbolic container that supports affect modulation while reducing the risk of emotional overload [35,36]. The visual–plastic mediation characteristic of Art Therapy allows patients to externalize internal states without direct bodily activation, which may be particularly advantageous in individuals with psychosis vulnerability, for whom intense sensorimotor stimulation can be destabilizing [25]. The comparatively lower effectiveness of DMT observed in this group may therefore reflect the greater complexity and potential destabilizing effects of direct bodily engagement in early stages of psychosis, underscoring the importance of carefully matching therapeutic modality to clinical profile and stage of illness [24].

### 4.4. Clinical Implications

Taken together, the present findings suggest that Creative Expressive Therapies should not be implemented as uniform interventions but rather applied according to principles of clinical appropriateness and individualization. Dance/Movement Therapy may be particularly suited to conditions characterized by prominent somatic dysregulation and disturbances in body representation, such as EDs, whereas Art Therapy may be more appropriate in clinical contexts in which affect regulation and symbolic organization represent primary therapeutic targets. From a translational perspective, these results support the integration of Creative Expressive Therapies within multidisciplinary mental health services as mechanism-informed adjunctive interventions, tailored to predominant dimensions of dysfunction rather than diagnostic categories alone. Such an approach is consistent with emerging calls for more personalized and function-oriented models of mental health care within public healthcare systems [37].

### 4.5. Limitations

Several limitations of the present study should be acknowledged. First, the quasi-experimental design and the non-randomized allocation of participants limit the ability to draw causal inferences regarding the comparative effectiveness of Art Therapy and Dance/Movement Therapy and may have introduced selection bias related to clinical indication. Second, the relatively small sample size, particularly within each clinical subgroup, may have reduced statistical power and limits the generalizability of the findings, especially with respect to interaction effects. Third, outcome assessment relied on ad hoc self-report measures that, although demonstrating good internal consistency, have not undergone formal validation and therefore warrant caution in the interpretation of effect magnitude. Furthermore, the use of an ad hoc questionnaire represents an additional limitation. Although the instrument was designed to capture clinically relevant dimensions of emotional and somatic regulation, its psychometric properties have not yet been formally validated, limiting direct comparability with other studies. Although large effect sizes were observed, these reflect within-sample changes in experiential and embodied process measures rather than estimates of clinical efficacy or symptom reduction. Accordingly, findings should be interpreted as hypothesis-generating signals to inform future controlled trials. In addition, the absence of long-term follow-up precludes conclusions regarding the durability of the observed effects over time. Finally, the pragmatic clinical setting, while enhancing ecological validity, may have introduced unmeasured confounding factors. Future research should address these limitations through adequately powered randomized controlled trials, the use of validated outcome measures, and longitudinal designs to clarify the specificity and clinical relevance of Creative Expressive Therapies. In addition, the small subgroup sample sizes raise concerns regarding the robustness of higher-order interaction effects. In particular, the three-way Treatment × Clinical Group × Time interactions should be interpreted with caution due to the increased risk of overfitting and Type I error inflation. These findings should therefore be considered preliminary and hypothesis-generating rather than confirmatory.

Accordingly, the present findings should not be interpreted as evidence of treatment efficacy, but rather as preliminary indications of differential response patterns that warrant further investigation in controlled, adequately powered trials. Future research should prioritize randomized controlled designs, larger and adequately powered samples, longitudinal follow-up assessments, and the use of standardized and validated outcome measures to test the preliminary patterns identified in this exploratory study.

## 5. Conclusions

This exploratory study suggests that Creative Expressive Therapies are associated with improvements in perceived well-being as well as emotional and physical tension regulation when integrated into standard care for individuals with FEP and EDs. The findings further indicate potential modality-specific patterns of response, with Dance/Movement Therapy showing relatively greater effects on somatic regulation in EDs and Art Therapy demonstrating relatively greater effects on emotional regulation in FEP. These results should be interpreted with caution, given the non-randomized design, the small sample size, and the use of ad hoc self-report measures. Accordingly, the present study is best considered hypothesis-generating rather than confirmatory. Future research should employ adequately powered randomized controlled designs, validated outcome measures, and longitudinal follow-up to clarify the robustness, specificity, and clinical relevance of these preliminary findings. Within these limitations, the present work contributes to the growing body of literature supporting a more individualized and function-oriented application of Creative Expressive Therapies in mental health care, emphasizing the importance of aligning therapeutic modality with predominant dimensions of clinical dysfunction. Taken together, these findings support further investigation of Creative Expressive Therapies as potentially differentiated interventions across clinical populations, rather than definitive conclusions regarding their comparative effectiveness.

## Figures and Tables

**Table 1:** Sample characteristics and group allocation by clinical condition and intervention.

Group	Clinical Condition	Intervention	N
G1	FEP	Dance/Movement Therapy	7
G2	FEP	Art Therapy	7
G3	EDs	Dance/Movement Therapy	11
G4	EDs	Art Therapy	11
Total			36

Note: EDs: Eating Disorders, FEP: First Episode Psychosis.

**Table 2:** Outcome variables, clinical constructs, and measurement tools.

Dependent Variable	Clinical Construct	Measurement Tool
Perceived Well-Being	Subjective evaluation of mood and quality of life	Self-administered questionnaire (ad hoc)
Emotional Tension Regulation	Perceived ability to recognize, tolerate, and regulate intense emotional states	Self-administered questionnaire (ad hoc)
Physical Tension Regulation	Awareness of bodily sensations and ability to modulate somatic tension and anxiety-related physical states	Self-administered questionnaire (ad hoc)

**Table 3:** Sample characteristics and psychometric properties of the outcome measures.

Variable/Scale	Detail/Statistic	Value
Sample size (N)	Total participants	36
Age (years)	Mean ± SD	17.0 ± 2.0
Gender distribution	Females	30 (83.3%)
	Males	6 (16.7%)
U1—Perceived Well-Being	Cronbach’s alpha	0.88
U2—Emotional Tension Regulation	Cronbach’s alpha	0.84
U3—Physical Tension Regulation	Cronbach’s alpha	0.81

**Table 4:** Repeated-measures ANOVA results (Time factor: Session 1 vs. Session 10).

Dependent Variable	Mean S1 (SD)	Mean S10 (SD)	F(1, 32)	*p*-Value	η^2^*p*
U1—Perceived Well-Being	28.58 ± 5.1	41.67 ± 5.4	142.12	<0.001 *	0.81
U2—Emotional Tension Regulation	27.61 ± 5.4	38.08 ± 5.2	118.45	<0.001 *	0.78
U3—Physical Tension Regulation	28.67 ± 5.8	39.06 ± 5.5	105.73	<0.001 *	0.76
Dependent Variable	Mean S1 (SD)	Mean S10 (SD)	F(1, 32)	*p*-value	η^2^*p*

Note. η^2^*p* = partial eta squared. Values ≥ 0.14 indicate a large effect size. * *p* < 0.05.

**Table 5:** Linear mixed-effects models: Treatment × Clinical Group × Time interaction effects.

Interaction Predictor	Variable	Β	t-Value	*p*-Value	Interpretation
DMT × EDs × Time	U3—Physical Tension Regulation	2.82	1.98	0.019 *	Pattern of change consistent with hypothesized trend (H1b)
Art Therapy × FEP × Time	U2—Emotional Tension Regulation	1.57	2.45	0.026 *	Pattern of change consistent with hypothesized trend (H1c)
DMT × EDs × Time	U1—Perceived Well-Being	3.09	1.99	0.049 *	Pattern of change consistent with hypothesized trend (H1b)

Note. DMT = Dance/Movement Therapy; EDs = Eating Disorders; FEP = First-Episode Psychosis; U1 = Perceived Well-Being; U2 = Emotional Tension Regulation; U3 = Physical Tension Regulation; β = regression coefficient. Models included participant as a random intercept. * *p* < 0.05.

**Table 6:** Effect size magnitude (Cohen’s d) by clinical subgroup and intervention.

Clinical Group	Variable	Intervention	Cohen’s d	Effect Magnitude
EDs	U3—Physical Tension Regulation	DMT	0.92	Large
		Art Therapy	0.65	Medium
FEP	U2—Emotional Tension Regulation	Art Therapy	0.84	Large
		DMT	0.58	Medium

Note. EDs = Eating Disorders; FEP = First-Episode Psychosis; DMT = Dance/Movement Therapy; U2 = Emotional Tension Regulation; U3 = Physical Tension Regulation. Cohen’s d effect sizes were interpreted as follows: 0.20 = small, 0.50 = medium, 0.80 = large.

**Table 7:** Temporal trend analysis: Mean improvement across treatment phases.

Treatment Phase	Sessions	Characteristics of Improvement	Significance
Initial	1–3	Therapeutic latency/stabilization	*p* > 0.05
Intermediate	4–8	Peak clinical efficacy	*p* < 0.01 *
Final	9–10	Consolidation of outcomes	*p* < 0.05 *

Note. Trend analysis suggests a non-linear pattern of clinical change, with an initial latency phase followed by a significant increase and subsequent stabilization of treatment effects. * *p* < 0.05.

## Data Availability

The data presented in this study are available upon request from the corresponding author due to privacy and ethical restrictions related to sensitive clinical data.
